# Exploring Macroinvertebrate Species Distributions at Regional and Local Scales across a Sandy Beach Geographic Continuum

**DOI:** 10.1371/journal.pone.0039609

**Published:** 2012-06-25

**Authors:** Iván F. Rodil, Tanya J. Compton, Mariano Lastra

**Affiliations:** 1 Departamento de Ecología y Biología Animal, Universidad de Vigo, Vigo, Spain; 2 National Institute of Water and Atmospheric Research, Hamilton, New Zealand; Technical University of Denmark, Denmark

## Abstract

Exposed sandy beaches are highly dynamic ecosystems where macroinvertebrate species cope with extremely variable environmental conditions. The majority of the beach ecology studies present exposed beaches as physically dominated ecosystems where abiotic factors largely determine the structure and distribution of macrobenthic communities. However, beach species patterns at different scales can be modified by the interaction between different environmental variables, including biotic interactions. In this study, we examined the role of different environmental variables for describing the regional and local scale distributions of common macrobenthic species across 39 beaches along the North coast of Spain. The analyses were carried out using boosted regression trees, a relatively new technique from the field of machine learning. Our study showed that the macroinvertebrate community on exposed beaches is not structured by a single physical factor, but instead by a complex set of drivers including the biotic compound. Thus, at a regional scale the macrobenthic community, in terms of number of species and abundance, was mainly explained by surrogates of food availability, such as chlorophyll a. The results also revealed that the local scale is a feasible way to construct general predictive species-environmental models, since relationships derived from different beaches showed similar responses for most of the species. However, additional information on aspects of beach species distribution can be obtained with large scale models. This study showed that species-environmental models should be validated against changes in spatial extent, and also illustrates the utility of BRTs as a powerful analysis tool for ecology data insight.

## Introduction

Exposed sandy beaches dominate the world’s open coastlines shaping one of the most valuable coastal ecosystems. Increasing recreational and financial demands are placing escalating pressures on sandy beaches at unprecedented scales [1], [2]. Although exposed beaches have been considered marine deserts, they are teeming with life that provides numerous goods and services [3]. Specifically, macrobenthos are an integral component of beaches that process large quantities of organic material, recycle nutrients, and are also a fundamental food resource for higher vertebrates of both, commercial importance and conservation significance [3], [4].

Macroinvertebrates in exposed beaches show specific adaptations to the dynamic environment provided by the interactions of sand, waves, and tides, also called beach morphodynamics [3]. An understanding of the relationships between beach morphodynamics and the macroinvertebrate community has been critical in beach ecology studies [4]–[8]. Studies on beach macroinvertebrate have mainly focused on the elucidation of spatial and temporal patterns along a gradient of contrasting morphodynamic types from harsh reflective (coarse sands, steep slopes) to benign dissipative (fine sands, gentle slopes) beaches [3], [4], [6], [7], [9]. These studies showed that physical variables inevitably drive the composition of the macroinvertebrate community. Therefore, the number of macroinvertebrate species, their abundance and/or their biomass, generally increases towards the more benign morphodynamic beach type [4]. Beach type thus represents a dynamic spectrum of environmental gradients where both, local and regional factors constrain and modify macroinvertebrate distributions. However, the processes that explain macroinvertebrate species distribution patterns across different spatial scales using several morphodynamic conditions and beach characteristics have not been examined.

There are a number of environmental and biological variables that influence the distribution and abundance of beach macroinvertebrates, including sediment grain size, hydrodynamic regime, biotic interactions and food supply [6], [7], [10]–[12]. Some of these variables affect beach species patterns at a local scale (e.g. slope), other variables have an influence over a broader scale (e.g. food availability); while other variables have both local and large scale effects (e.g. sediment characteristics). Thus, species-environment relationships can change with the scale of observation, because at different spatial scales different processes may influence species distributions [13]. For example, a species may be locally limited to beaches with fine grain sizes, but at a larger geographical scale species can also be limited by other variables, such as food availability. Therefore, over a broad spatial scale, the same species may show preferences for beaches with coarser sediment types, but also beaches that are food-enriched. The analysis of scale dependent relationships can help to distinguish whether specific variables are direct drivers of species patterns or are mediated by other variables [13].

In this study, we examined the role of different environmental variables for describing the regional and the local scale distributions of representative macrobenthic species across 39 beaches along the North coast of Spain. This shoreline is characterised by extensive, intermediate morphodynamic beaches that differ in size, orientation and physical characteristics across more than 2000 km of coastline. This large continuum of beaches provides an opportunity to assess the drivers of species distributions at contrasting-scales, i.e., regional versus local responses of macroinvertebrates over a gradient of intermediate beaches. To identify which environmental variables explain the regional distribution of the macrobenthic species we used estimates of chlorophyll a, temperature and morphodynamic variables that broadly characterise a beach. We expect that at a regional scale, the macroinvertebrate abundance and richness would follow a longitudinal gradient related to primary productivity [14]. On the other hand, for each beach a specific abiotic variable might be the driver limiting or shaping a local species response. To test whether the responses of individual species to the same environmental variable differ at a local scale, we examined the fitted responses of species within a beach to local variables (e.g., slope, sediment).

Different techniques have been used for testing beach species environment relationships (e.g., [4], [6], [7], [15], [16]), but these have mainly focused on contrasting morphodynamic gradients at a single spatial scale. To examine the role of environmental variables for describing macroinvertebrate species distributions at different spatial scales we used gradient-boosted regression trees (BRTs). BRT stems from machine learning [17], and can automatically model complex functions and the interactions between variables without making assumptions about the shape of the fitted functions or the interactions between variables [18], [19]. It thus provides a powerful tool for analysing complex ecological datasets, and examining the distribution of beach species over different spatial scales.

## Methods

### Study Site and Sampling Procedure

**Figure 1:**
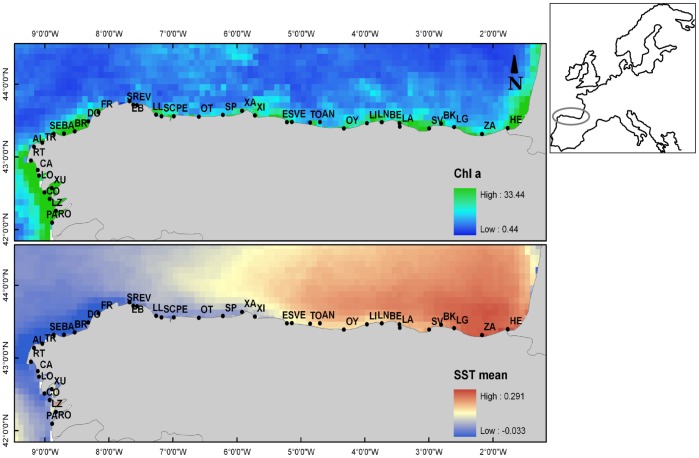
The Spanish northern shoreline, showing the location of the 39 beaches. (from East to West; HE: Hendaya; ZA: Zarautz; LG: Laga; BK: Bakio; SV: Salvaje; LA Laredo; BE: Berria; LN: Langre; LI: Liencres; OY: Oyambre; AN: Andrín; TO: Toranda; VE: Vega; ES: Espasa; XI: Xivares; XA: Xagó; SP: San Pedro; OT: Otur; PE: Peñarronda; SC: San Cosme; LL:Llás; EV: Viveiro; SR: San Román; EB: Bares; FR: Frouxeira; DO: Doniños; BR: Barrañán; BA: Baldaio; SE: Seiruga; TR: Traba; AL: Area Longa; RT: Rostro; CA: Carnota; LO: Louro; XU: Xuño; CO: Corrubedo; LZ: Lanzada; RO: Rodas; PA: Playa América). Estimates of mean sea surface temperature (sstmean) and chlorophyll a (chla) concentrations are shown.

Thirty nine exposed sandy beaches were sampled from 1995 to 1999 along the North coast of Spain (Figure 1). Sampling was carried out during spring tides in September and October to reduce biotic and abiotic variability linked to seasonal cycle in exposed beaches [7], [8], [14]. This coast encompasses *ca*. 2000 km of shoreline between 42° 07′N, 1° 46′ W and 43° 22′ N, 8° 49′ W (data compiled from [8], [14], and Rodil unpublished data). All beaches are mesotidal, with maximum tidal ranges close to 4 m. This coast is affected by upwelling events along the North margin of the Iberian Peninsula (from West to East) stimulating primary production during the spring and summer seasons [20], related to cold sea water temperature and high chlorophyll a concentrations (Figure 1).

At each beach, macroinfauna samples were collected at 10 equally spaced intervals (distance variable depending on the total width of each beach) from above the drift line to the low swash zone with one 0.05 m^2^ core per interval to a depth of 15 cm (see [8], [14] for further details). All macroinvertebrate samples were taken along three randomly chosen transects during low tide. A total of 1170 macrobenthos samples were collected in the study area. All the sediment samples were sieved through 1-mm mesh and the residue was preserved in 4% formalin. The individuals were later sorted from the sediments, identified and counted in the laboratory. We obtained abundance (individuals/m^2^) and species richness (number of species) as the main beach macroinvertebrate community descriptors. Sediment samples for the determination of the granulometric measures and morphodynamic beach characteristics were collected in triplicate at the same sites as the macroinvertebrates (see supporting Table S1 for a detailed explanation of all the environmental variables). No specific permits were required for the described field studies.

**Figure 2:**
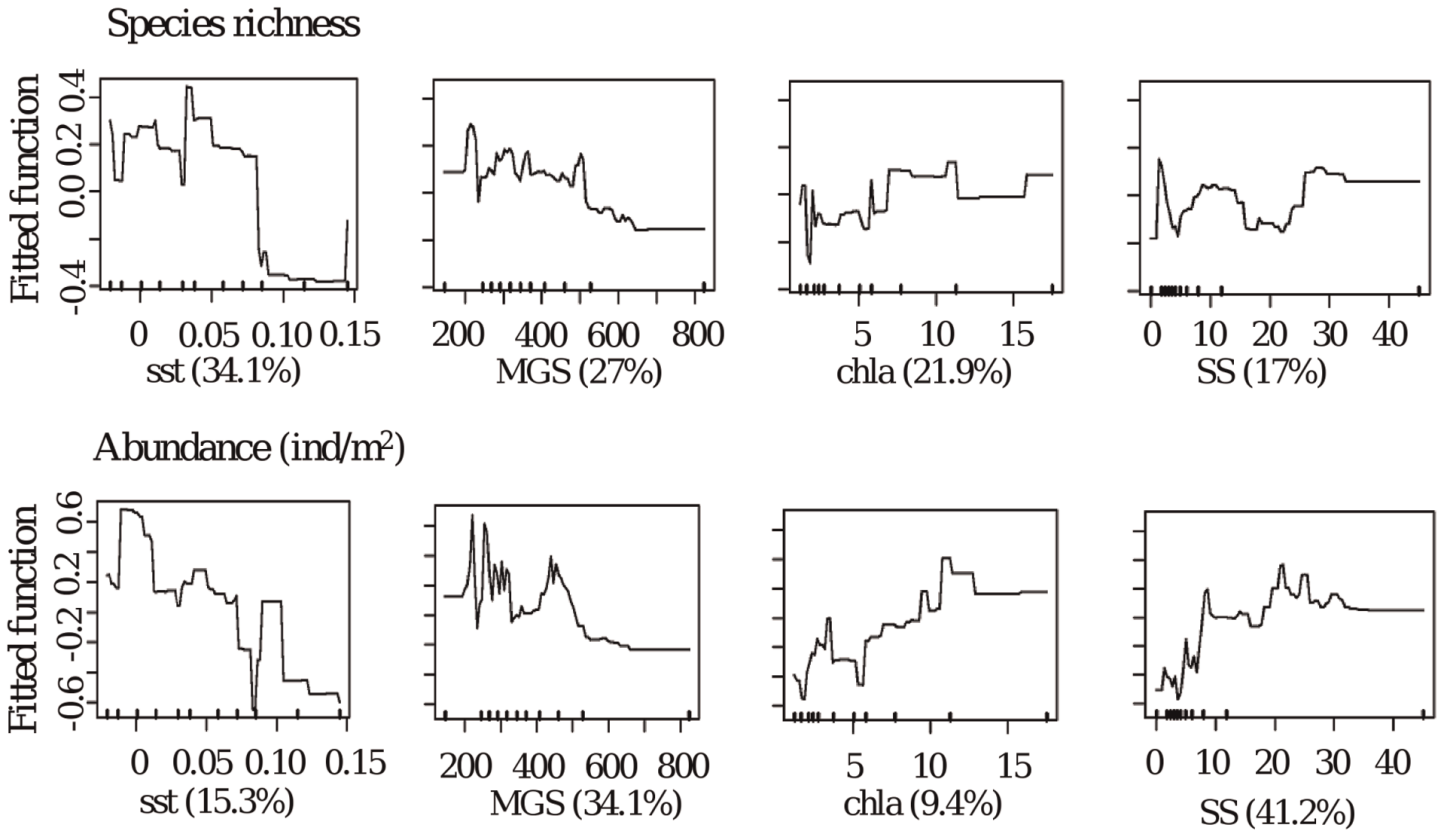
Variation in species richness and macroinvertebrate abundance (ind/m^2^) predicted by a BRT model. The relative importance of the most influential variables is shown as %. Ticks across the bottom of each plot show the distribution of deciles for each predictor variable. A common scale is used on the vertical axis (centred to have zero mean over the data distribution). Mean grain size (MGS), shear strength (SS), chlorophyll a (chla) and mean sea surface temperature (sstmean).

The beach environment from the northern coast of Spain comprises a full set of environmental variables (see Table S1). Typical beach morphodynamic indices, such as Dean’s parameter (Ω), Beach State Index (BSI) and Beach Index (BI) [3] were used to characterize the beach morphodynamic type (see Table S1 for an index description). These indices classified all the beaches as a series of intermediate tidally dominated environments (92% and 97% of the beaches according to Ω and BSI, respectively); considered the most varied and widespread type of beach worldwide [3] falling between the two main morphodynamic extremes (reflective and dissipative). The rating system proposed by [21] was used to estimate the wave exposure rate (ER, Table S1). Estimates of average sea surface temperature and chlorophyll a concentrations were obtained from Bio-Oracle (http://www.oracle.ugent.be/) [22]. Specifically, annual average chlorophyll a (chla) and sea surface temperature (sst), proxies of seasonality and temporal variation in nutrient supply, were derived from satellite images (Aqua-MODIS and SeaWiFS; http://oceancolor.gsfc.nasa.gov/) summarizing information across several years and were then used to generate three relevant metrics: annual maximum, minimum and mean (Figure 1). The estimates of chla and sst were extracted from the GIS layers to the sample sites in this study using the intersect points tool from Hawths Tools in ArcGIS 9.2. These two metrics showed a specific geographic gradient with decreasing temperature and high chla in waters from East to West (Figure 1).

**Figure 3:**
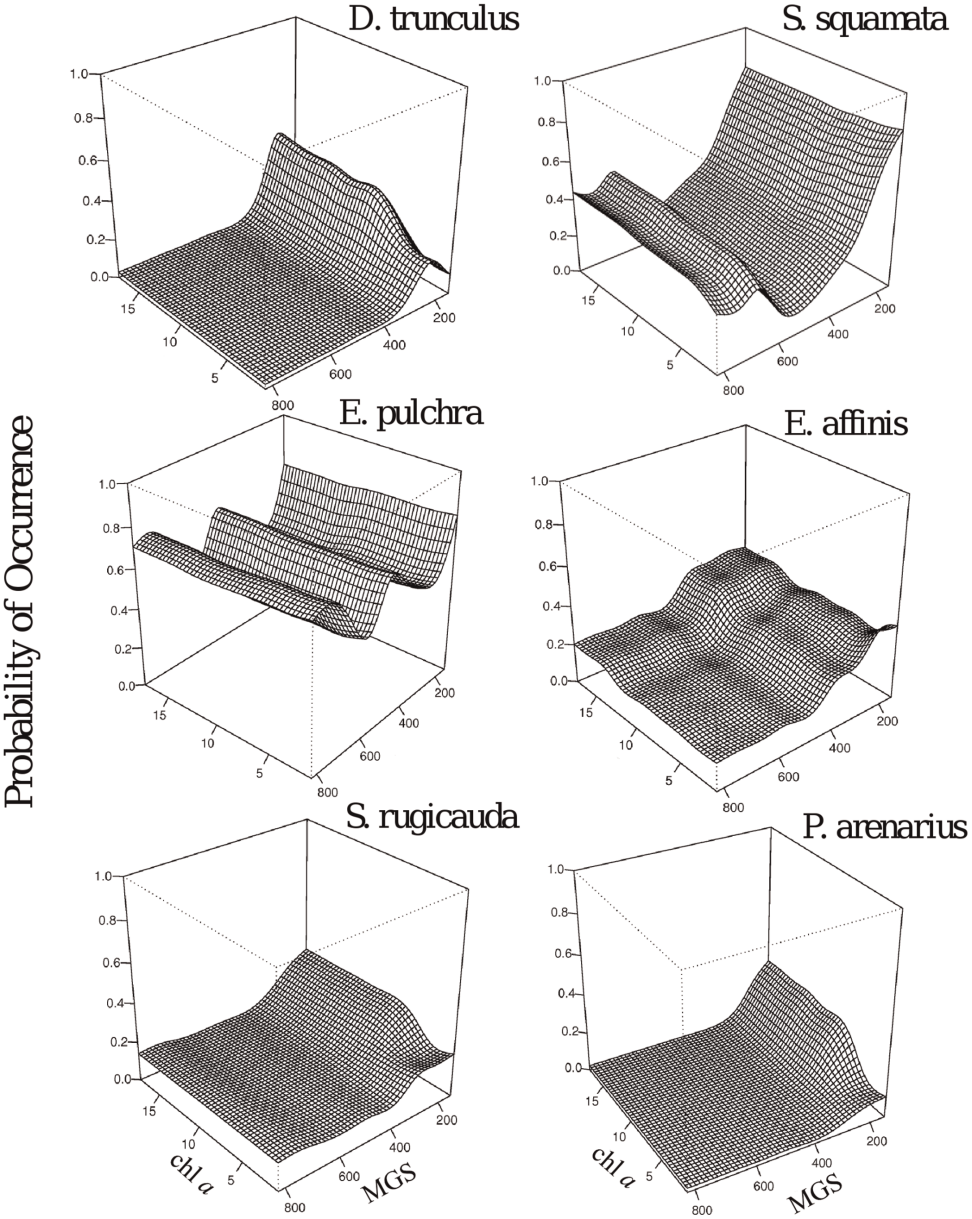
Three-dimensional partial dependence plots. Plots show the strongest pairwise interactions in the beach model for the main species (*Donax trunculus*, *Scolelepis squamata*, *Eurydice pulchra*, *Eurydice affinis*, *Sphaeroma rugicuda*, *Pontocrates arenarius*). Mean grain size (MGS), and chlorophyll a water concentration (chl*a*) as the main predictor variables.

**Table 1:** Predictive performance of the BRT model relating the beach species distributions to environment, averaged across the species (presence/absence) over the regional scale.

					Cross-validation*			
Main species	pa	lr	n.trees	Null deviance	Unexplained deviance (se)	Explained deviance (se)	AUC (se)	Correlation (se)
*B. pelagica*	78	0.001	1250	0.490	0.439 (0.01)	10.3 (0.03)	0.751 (0.03)	0.257 (0.04)
*D. trunculus*	88	0.001	1200	0.534	0.446 (0.03)	16.4 (0.05)	0.815 (0.03)	0.325 (0.06)
*E. affinis*	213	0.001	1200	0.949	0.877 (0.01)	7.6 (0.01)	0.709 (0.02)	0.276 (0.02)
*E. pulchra*	588	0.003	1000	1.386	1.356 (0.01)	2.2 (0.01)	0.6 (0.01)	0.176 (0.02)
*P. arenarius*	332	0.001	1050	1.193	1.146 (0.01)	4 (0.01)	0.636 (0.02)	0.22 (0.03)
*S. squamata*	368	0.001	1700	1.245	1.164 (0.01)	6.5 (0.01)	0.677 (0.01)	0.284 (0.02)
*S. rugicauda*	186	0.0003	2150	0.876	0.857 (0.01)	2.2 (0.01)	0.61 (0.02)	0.147 (0.02)
*T. brito*	49	0.000	1350	0.348	0.338 (0.01)	2.7 (0.02)	0.661 (0.04)	0.106 (0.03)
*T. saltator*	61	0.0003	1050	0.410	0.406 (0.01)	1 (0.01)	0.608 (0.04)	0.068 (0.03)

* Mean and se estimated within model building. Standard error (se).

Estimates of predictive performance were calculated using 10-fold cross validation and a tree complexity of 3. Learning rate (lr), the number of trees (n.trees), and the number of positive observations (pa) are shown. AUC: area under the receiver operating characteristic curve. The final environmental variables included in the models are shown in Table 2. Beach species included in the final model: *Bathyporeia pelagica*, *Donax trunculus*, *Eurydice affinis*, *Eurydic pulchra*, *Pontocrates arenarius*, *Scolelepis squamata*, *Sphaeroma rugicauda*, *Talorchestia brito* and *Talitrus saltator*.

**Table 2:** The relative contribution (%) of the predictor variables to the BRT models over the regional scale.

Main species	MGS	SS	sst	chla
*B. pelagica*	60.4	17.7	11.2	10.7
*D. trunculus*	53.5	31.9	4.5	10.1
*E. affinis*	40.1	47.7	3.8	8.3
*E. pulchra*	50.6	35	9.7	4.7
*P. arenarius*	20.7	64	11.1	4.1
*S. squamata*	52	34.6	6.2	7.1
*S. rugicauda*	60.1	21.1	7	11.9
*T. brito*	17.5	60.5	12.8	9.2
*T. saltator*	39.4	40.7	12.2	7.6

The predictor variables, by relevance were mean grain size (MGS), sediment shear strength (SS), mean sea surface temperature (sst), and chlorophyll *a* (chl*a*). The predictor variables with the highest contribution described the recorded observations of the dominant beach species (same as in Table 1). Beach species included in the final model: *Bathyporeia pelagica*, *Donax trunculus*, *Eurydice affinis*, *Eurydic pulchra*, *Pontocrates arenarius*, *Scolelepis squamata*, *Sphaeroma rugicauda*, *Talorchestia brito* and *Talitrus saltator*.

All variables (Table S1) were checked for collinearity using a Pearson correlation. Where the correlation was greater than 0.7, the variable within the pair that had the best functional relevance (i.e., the most important morphodynamic influence according to the literature) of the variables was kept in the model.

### Machine Learning Analyses

We used boosted regression trees analysis (BRT) to examine the association between total macroinvertebrate abundance and species richness, with respect to variables describing the beach environment across the northern Spanish coastline. BRT, a machine learning approach [17], [23], combines the regression tree algorithm and the boosting algorithm to produce an ensemble of regression trees. The boosting algorithm improves standard regression tree modelling by adding a stochastic component to the model, which continuously emphasizes the poorly explained part of the data space [17], [19]. BRT can be considered an advanced form of regression, which also uses a link function to examine a range of response types including binomial, Poisson and Gaussian [24]. Although the ecological uses of BRT have only been recently applied, it has shown to be a useful technique for dealing with complex biological data sets [25]–[28].

**Table 3:** Comparison of the predictive performance of statistical models relating dominant species distributions to environment, averaged for the beach species in a local scale.

					Cross-validation*		
Main species	pa	lr	n.trees	Null deviance	Unexplained deviance (se)	Explained deviance (se)	Correlation (se)
*B. pelagica*	1170	0.0003	2200	3.90	3.1 (0.7)	0.2 (0.1)	0.331 (0.11)
*D. trunculus*	1170	0.001	3650	4.02	2.4 (0.3)	0.41 (0.09)	0.612 (0.06)
*E. affinis*	1170	0.005	2000	7.07	2.6 (0.2)	0.64 (0.03)	0.71 (0.08)
*E. pulchra*	1170	0.005	7300	18.52	6.7 (1.1)	0.64 (0.06)	0.764 (0.06)
*P. arenarius*	1170	0.005	2700	3.40	2.1 (0.15)	0.4 (0.04)	0.531 (0.04)
*S. squamata*	1170	0.005	5100	8.87	3.1 (0.4)	0.65 (0.05)	0.745 (0.04)
*S. rugicauda*	1170	0.005	1450	3.25	2.5 (0.5)	0.23 (0.1)	0.46 (0.06)
*T. brito*	1170	0.001	1900	5.72	3.6 (1.8)	0.4 (0.3)	0.614 (0.13)
*T. saltator*	1170	0.0001	1600	1.89	1.5 (0.3)	0.2 (0.17)	0.631 (0.06)

* Mean and SE estimated within model building. Values indicate the cross-validated unexplained and % explained deviances, and the cross-validated correlation (cvCor) and the standard errors (se).

Estimates of predictive performance were calculated using 10-fold cross validation and a tree complexity of 3. Learning rate (lr), the number of trees (n.trees), and the number of positive observations (pa) are shown. Environmental variables tested in the final model are showed in Table 4. Beach species included in the final model: *Bathyporeia pelagica*, *Donax trunculus*, *Eurydice affinis*, *Eurydic pulchra*, *Pontocrates arenarius*, *Scolelepis squamata*, *Sphaeroma rugicauda*, *Talorchestia brito* and *Talitrus saltator*.

The predictive performance of the BRT models is optimized by means of the learning rate (lr) and tree complexity (tc). The lr is used to shrink the contribution of each tree as it is added to the model, and tc determines the number of nodes in a tree and should reflect the true interaction order on the response being modelled [23]. All BRT models had a tc of 3 and were optimised for their lr so that a minimum of 1000 trees was fitted for each model [19]. Model performance was assessed using ten-fold cross-validation (cv). This procedure compares fitted values from ten individual models, each derived from a random subset of the full data, against the portion of the data withheld from the model [29]. Interactions between predictors are modelled automatically with BRT because the structure of a tree means that the response to one predictor variable depends on values of predictors higher in the tree [27]. All BRT analyses were carried out in R (version 2.14.0) [30] using the ‘gbm’ library [31], supplemented with functions from [19].

**Table 4:** The relative contribution (%) of the predictor variables to the BRT interaction models over the local scale.

Main species	Beach	MGS	Slope	SS
*B. pelagica*	54.9	38.3	2.7	4.1
*D. trunculus*	28.6	17.3	31.8	22.3
*E. affinis*	27.6	10.9	37.7	23.9
*E. pulchra*	31.7	20.2	13.8	34.3
*P. arenarius*	34.4	13.7	38	13.8
*S. squamata*	50.5	9	29.5	11
*S. rugicauda*	42.6	18.7	29.1	9.6
*T. brito*	0.7	31.1	63.2	5
*T. saltator*	0.2	1.3	97.2	1.3

The predictor variables were Beach, mean grain size (MGS), beach slope, and sediment shear strength (SS). The predictor variables with the highest contribution described best the recorded observations of the main beach species. Beach species included in the final model: *Bathyporeia pelagica*, *Donax trunculus*, *Eurydice affinis*, *Eurydic pulchra*, *Pontocrates arenarius*, *Scolelepis squamata*, *Sphaeroma rugicauda*, *Talorchestia brito* and *Talitrus saltator*.

### Regional Scale Analysis

Across all the 39 beaches, variation in macroinvertebrate abundance and species richness, and the occurrence of specific species was examined with respect to six predictor variables: Dean morphodynamic index (Ω), exposure rate (ER), sediment mean grain size (MGS) and shear strength (SS), sea surface temperature (sst), and chlorophyll a (chla) (see Table S1). Morphodynamic indices, and also ER are traditionally used to make beach comparisons [3], rather than to find different results within a beach. As sediment characteristics (MGS and SS) can show an effect over different spatial scales, they were included in both, the local and regional BRT models.

First of all, we explored the variation in species richness and macroinvertebrate abundance with respect to the beach variables across the geographic gradient using a BRT model with a Poisson link function. We further examined the distributions of the most common macroinvertebrate species at beaches along the northern Spanish coastline using a BRT model with a Bernoulli link function to fit the presence/absence response with respect to the predictor variables. Model performance was examined with the area under the receiver-operating characteristic curve (AUC) [32]. The AUC provides a measure of the degree to which the fitted values discriminate the observed presences and absences, where a value of 1 indicates that the presences and absences were perfectly discriminated while a value of 0.5 indicates that a model cannot discriminate the presences better than random.

**Figure 4:**
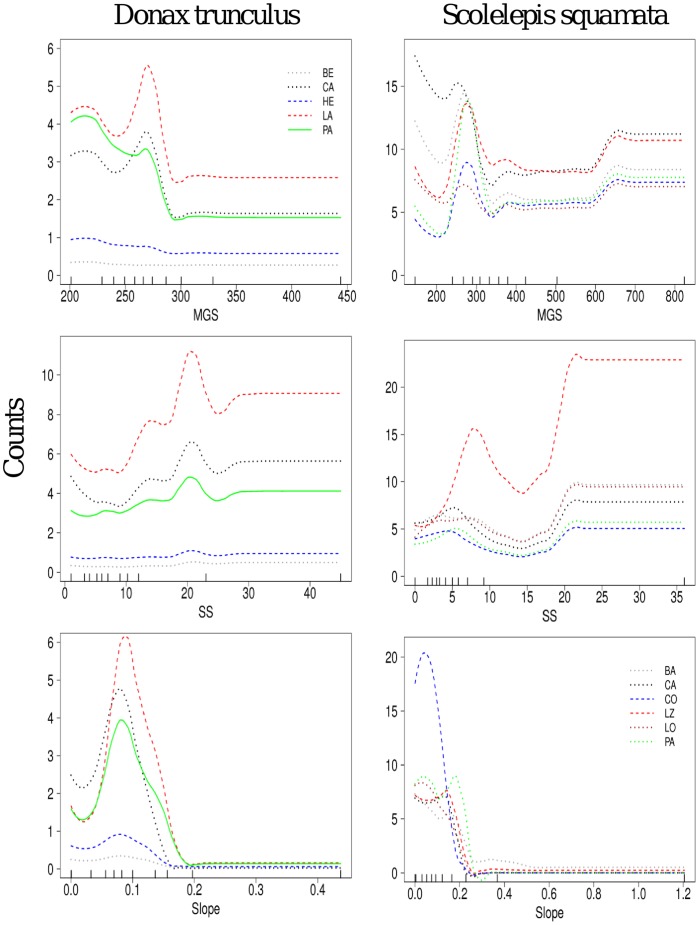
The abundance of *D. trunculus* and *S. squamata* predicted by the main beach variables. The species abundance (counts) was plotted over those beaches presenting a frequency >5 individuals. Ticks across the bottom of each plot show the distribution of deciles for each predictor variable. Main variables within a beach: mean grain size, shear sediment strength, and slope. Beach names as in Figure 1.

**Figure 5:**
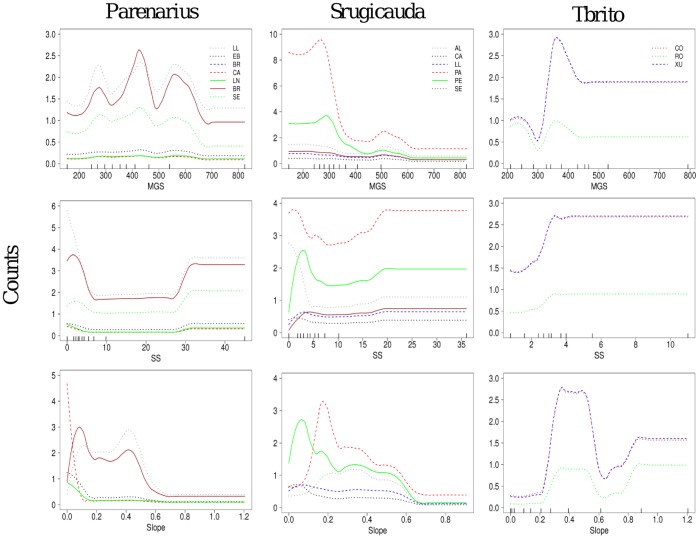
The abundance of *Pontocrates arenarius*, *Sphaeroma rugicauda* and *Talorchestia brito* predicted by the main variables. The species abundance (counts) was plotted over those beaches presenting a frequency >5 individuals. Predictor variables names as in Figure 4. Ticks across the bottom of each plot show the distribution of deciles for each predictor variable. Beach names as in Figure 1.

**Figure 6:**
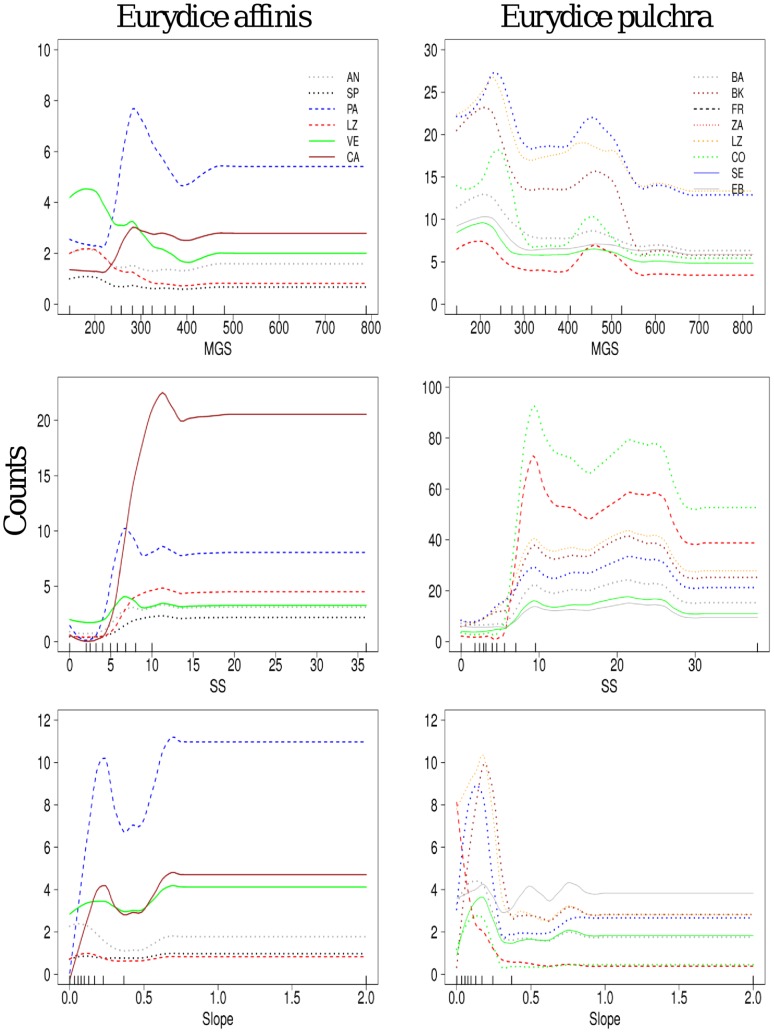
The abundance of *E. affinis* and *E. pulchra* predicted by the main beach variables. The species abundance (counts) was plotted over those beaches presenting a frequency >5 individuals. Predictor variables names as in Figure 4. Ticks across the bottom of each plot show the distribution of deciles for each predictor variable. Beach names as in Figure 1.

To assess the relevance of each variable to the BRT models, the variable contribution was used. This variable gives a measure of how often the predictor is selected, and the improvement to the model as the result of a variable being selected [27]. This measure assesses the number of times a variable is selected for splitting, weighted by the squared improvement to the model as a result of each split, and averaged over all trees [19]. The relative contribution of each variable to a model was assessed using functions provided by [19]. In the cases where the predictor variables played a small contribution (<3%) to explaining variation in the final BRT model, these variables were eliminated from the final models. The probability of occurrence at a regional scale was examined using perspective plots ([19], gbm.perspec).

### Local Scale Analysis

At a local scale, the species distributions were examined with respect to exposure time (ET), sediment water content, slope, sediment characteristics (MGS and SS), sediment temperature, and Beach (Table S1). Beach is added as a factor in the BRT models, to determine whether individual species distributions displayed different responses between the different beaches. Note that in this analysis we added the condition that recorded observations had to exceed an N = 5 before a species at a particular beach could be included in this analysis, thus not all 39 beaches in the regional analysis ended up in the species models for the local analysis. Less than N = 5 would mean that we don’t have enough data to describe the distribution of a species with respect to an environmental gradient.

To identify whether individual species had different environmental associations among beaches, we examined the abundance of the most common species (in terms of abundance and occurrence; Table S2, supporting information) with respect to the environmental variables using a BRT model with a Poisson link function. This model represented the data better than a log-normal model with a Gaussian error distribution. The model performance was assessed using the cross-validated (cv) explained deviance, which provides a measure of the goodness-of-fit between the predicted and raw values, and the cross-validated correlation (cvCor), which provides a measure of correlation between the recorded observations and the model fitted values. The cvCor is calculated as a Pearson correlation coefficient and thus takes into account how far the prediction varies from the observed data [33].

To visualize the fitted functions from the local BRT model, partial dependence plots were used ([19], gbm.unipred). These functions show the effect of a variable on the response, while controlling for the average effect of all other variables in the model [19]. The partial dependence plots for the most important predictors of the main species were presented, and interactions were explained in the text.

## Results

The environmental predictors from the 39 studied beaches displayed different physical and morphodynamic characteristics (Figure S1, supporting information) along the Spanish coastline (Figure 1), but there was no significant trend in harshness or a shift in the morphodynamic conditions across the geographic stretch. Sediment temperature was significantly related to beach location (Figure S1), with lower sediment temperatures at the most western beaches (y = 25.2–0.02x; R^2^ = 0.47, p<0.001). There were strong correlations (>0.7) between some of the main environmental variables, such as MGS, with sediment water content, Dean and ER. In this case, only MGS was selected for the final BRT regional models (including MGS, SS, chla and sst). Sediment water content and ET were highly correlated with beach slope, thus ET was excluded from the final local model (including MGS, SS, slope and beach) (see Table S1 for code explanation).

A total of 71 species were collected through all the 39 beaches. Macroinvertebrate community was dominated by Crustacean species (41%), Isopoda and Amphipoda being the main components of this group (71%). Polychaetes and molluscs were also present but in lower percentages (31 and 3%, respectively). In the supralittoral zone, a variety of insects (13 species) were found above the drift line (18%). Five different species (7%) belonging to different groups (one nemertean, one oligochaeta and three fish species) were also found (Table S3, supporting information).

### Regional Scale

Using 4 environmental variables to describe the beach conditions across the northern Spanish coastline, the BRT models described species richness (0.51±0.02 cvCor) and macroinvertebrate abundance (0.48±0.07 cvCor) with good predictive performances (Figure 2). Furthermore, the models explained 77.1 and 66.6% of the cross validated deviance for species richness and abundance, respectively. Inspection of the contributions of the predictor variables indicated that sst (34.1%), MGS (27%), chla (21.9%) and SS (17%) were the most important variables for explaining species richness (Figure 2). Species richness tended to increase with increasing chla and to decrease with increasing MGS, SS and sst (Figure 2). The variables most important for macroinvertebrate abundance were sediment shear strength (41.2%) and MGS (34.1%). Abundance was also related to high chla (9.4%) and low sst (15.3%) (Figure 2).

The BRT regional model showed a good performance for all the species (cvAUC >0.6), and for *B. pelagica*, *D. trunculus* and *E. affinis* (cvAUC >0.7) the model showed a very good degree of discrimination among the predictor variables (Table 1). The distribution of the dominant species along this coastline was described mainly by sediment variables (MGS and SS) and by chla and sst (Table 2). MGS explained the greatest amount of variability in describing the probability of occurrence across the beaches for *B. pelagica* (60.4%), *S. rugicauda* (60.1%) and *D. trunculus* (53.5%) (Table 2). However, sediment predictor variables were associated with chla (Figure 3). Thus, although the probability of occurrence of the most common species was higher with lower MGS, the fitted probabilities of occurrence were always associated with high chla concentrations (Figure 3). *E. pulchra* showed a wide geographic distribution across all the beaches with different probabilities of occurrence no matter the MGS-chla association (Figure 3).

### Local Scale

The abundance distribution of the most common species within a beach was explained with a high level of predictive performance (cvCor  = 0.3–0.76, Table 3), but some of the species had a relatively lower model performance (e.g. the model for *B. pelagica* explained only 20% of the predictive deviance, compared to 65% for *S. squamata*, or 64% for *E. affinis* and *E. pulchra*) (Table 3).

The predictor variables with the highest contribution to explaining the abundance distributions of individual species within the beaches are showed in Table 4. The sediment characteristics (MGS and SS) showed the highest overall contribution. The factor Beach was also an important variable for some species such as, *B*. *pelagica* (54.9%), *E. pulchra* (31.7%), *P. arenarius* (50.5%), *S. squamata* (42.6%), and *S. rugicauda* (34.4%). Other distributions of species, such as *T. brito* and *T. saltator* were explained by beach slope (63.2 and 97.2%, respectively) or MGS (*T. brito*, 31.1%) (Table 4).

The fitted responses of species to the predictor variables within a beach were generally repeatable between beaches (Figures 4, 5, 6). In some of the beaches not only was the shape of the predicted species response similar, but also the species abundance did not change very much between beaches (Figure 4, 5, 6). Thus, within the northern Spanish beaches, the bivalve *D. trunculus* was predicted to occur maximally in fine-grained sediments (MGS <300 µm) and low sediment shear strength (SS <25 Kpa) (Figure 4). *S. squamata* showed a similar sediment trend (Figure 4), as this polychaete also revealed maximum peaks of predicted abundance in fine-grained sediments (MGS <300 µm) and low sediment shear strength (SS <10 Kpa). *D. trunculus* and *S. squamata*, showed an expected higher occurrence in the lower parts of the beach face slope (<0.2; below the drift line). However, these species showed differences in the abundances between some of the beaches, with the highest abundances in the most western beach locations (Figure 4).

Some species, such as *P. arenarius*, and *S. rugicauda* showed a preference for low SS values (<10 Kpa), and a widespread preference for a wide range of grain sizes (Figure 5). These two species were found maximally in low intertidal levels (Slope ≤0.2) and, although with a similar fitted response to any environmental gradient, they showed different abundances between some of the beaches. The highest abundances were generally found in the western beaches (Figure 5). *T. brito* showed the highest peaks of abundance in fine-grain sizes (200–400 µm) and very low compacting sediment forces (<4 Kpa) (Figure 5). The highest predicted sand hopper abundances were found at the mid-high intertidal levels (Slope >0.3; above the drift line), and the relative contribution of SS (5%) and beach (1%) were low (Table 4).

Finally, the two sympatric cirolanid isopods *E. affinis* and *E.pulchra* showed repeatable response curves between beaches with respect to the main predictor variables (Figure 6); although they showed different abundances between some of the beaches. Both species were found more commonly in the lower parts of the intertidal (Slope <0.3), but also with some peaks of abundance in higher levels of the beach face slope (Slope >0.3, Figure 6). Interestingly, there was a contrasting response of the two species when the same variables were compared. *E. affinis* showed a specific association with low MGS (∼300 µm) and SS (5–10 Kpa). By contrast, *E. pulchra* showed an association with a broad range of median grain sizes (100–500 um), different levels of sediment compaction (5–30) and different slope environments (Figure 6), charting saw-shaped relationships with the environmental variables.

## Discussion

The distribution of the macroinvertebrate community is here explained with respect to 18 predictor variables across and within 39 beaches from the North coast of Spain. The current status and theories accept physical factors as the predominant predictor of community characteristics across beach types [3]–[9]. Gradually, however, a greater number of studies is showing the relevance of biotic interactions and food supply on exposed beaches (e.g., [11], [12], [14], [34]).

### Over a Large Scale: the Geographic Gradient

From a regional scale perspective, an ensemble of predictor variables including MGS, SS, and surrogates of food supply (chlorophyll a and sea surface temperature) showed the highest contribution to the models. Macroinvertebrate abundance and richness were higher where food supply was higher along the geographic gradient. Moreover, the spatial variation of the macroinvertebrate species reflects the extent to which other variables, apart from beach morphodynamics, shape the macroinvertebrate community from these locations. The contribution of the sediment characteristics to the dominant species from this region interacted with proxies of food availability, i.e., chla. This interaction was well described by the pairwise partial dependence plots, where the dominant species revealed a specific preference for finer sediments, but the highest probability of occurrence were always associated with chla, no matter the sediment traits. Thus, macroinvertebrate community was not subjected only to physical control, and those variables associated with the beach environment did not provide an exclusive description of macroinvertebrate distribution through the regional scale. This refers to a pattern in the NW coast of Spain that relates the low sea water temperature and high chla concentrations with periodic upwelling events, stimulating primary production and shaping a West to East gradient of food supply [14], [20]. Surprisingly, although macroinvertebrate species generally occur in the highly productive areas, they did not show differences in their type of response to the environmental predictors through the whole geographic continuum.

### In a Local Scale: from within a Beach

At a local scale, predictor variables such as sediment characteristics and beach face slope played the highest contribution to the models, performing almost an exclusive role explaining most of the macroinvertebrate community variation on the beaches. The response of the individual species to these predictor variables followed a repeatable pattern across a single predictor variable between beaches. Thus, the occurrence of truly marine species [3], such as *D. trunculus* or *S. squamata* increased in the lower parts of the beach (i.e., closer to the sea), and decreased with increasing slope (i.e., terrestrial conditions). On the other hand, a semi-terrestrial species, such as *T. brito* occurs mainly close to the higher parts of the beach, where normally finer sediments are also found. Sediment traits, such as MGS and SS described the observed beach occurrences, but the Beach itself played a main role explaining the species variation in the models. Since the responses of the species to the variables were similar, the relevance of beach as a predictor variable indicates differences in beach abundances. All these beaches are mesotidal, intermediate and show variable physical characteristics through the geographic continuum with no significant morphodynamic trends. The only difference among all the beaches was related to the increasing gradient of food supply, which can explain the differences in abundance among beaches.

### A Contrasting Scale-approach: Large vs. Local

One of the most interesting results was that the fitted occurrence of the most common species across an environmental gradient was similar between beaches (e.g., decreases, increases or unimodal). In some of the cases, although the species response to the predictor variable was similar, the absolute occurrence observed changed between beaches. For instance, the bivalve *D. trunculus* and the polychaete *S. squamata* showed different abundances among some of the beaches. There are no cues in our data showing a shift in the environmental severity over the geographic stretch, but polychaete and mollusc species are truly marine species and are expected to be more sensitive to the harsh beach climate. Therefore, these species may display specific adaptations to the beach swash climates [3], [4]. *D*. *trunculus* is a filter feeder depending on suspended matter and food particles in water. This suggests that, as scale moves through beaches at different longitudes, other factors become important overlaying any response to the main predictor variable. In the same way, *T. brito*, *P*. *arenarius* and *S. rugicauda* relationships with the variables shaped models with the same types of response curves, but with different abundances between some of the beaches. This might be due to complex interactions between life history, feeding, mobility, growth and reproduction, and other processes that would require detailed study to resolve. However, based on the history of upwelling events in this region we hypothesize that the food supply may affect the geographic abundance distribution of these species. This model type revealed that large-scale species-environment relationships arise from interactive processes between variables, rather than local responses to a specific abiotic variable being the dominant factor limiting distribution [13].

Exposed beaches in this coast are dominated by crustaceans performing tidal migrations throughout the entire beach looking for food, but also for a more stable habitat far away from the harsh wave climate [4], [12], [35], [36]. In our locations, despite the high abundance of talitrid amphipods and tylid isopods, BRT models were not able to predict their occurrence related to any of the physical variables, except for *T. brito*. The relationships indicated a strong influence of a specific predictor variable on local processes for this sand hopper, i.e., similar form of response and occurrence across scales. These semi-terrestrial species, although highly mobile, spend their whole life cycle in the supratidal zone, burrowed under arid conditions and performing nocturnal feeding (on wrack and carrion) migrations [36], [37]. They find a more stable environment in the supralittoral zone, which is independent of the dominant physical intertidal conditions [3], [4]. Thus, neither the beach nor the sediment traits showed a high contribution for talitrid species models. In general terms, this may reflect some biological processes like predation or feeding competition without relation to the abiotic variables included in the models [38], [39]. In fact, the availability of limiting resources in the beach ecosystem can potentially influence all these interactions, including cannibalism episodes reported for some talitrid species [40].

Some of the intraspecific differences found in this study might be the result of different densities and beach levels occupied, i.e., niche segregation, for some species, such as the conspicuous isopods *E. pulchra* and *E. affinis*. Both species display a tidal cycle of migration into the water column and dominated from mid tide level downwards [36], coping with the harsh beach conditions and remaining buried during daylight. *E. pulchra* was the most abundant species, inhabiting a broader intertidal spectrum and plotting a saw-shaped chart across the intertidal and over the geographic stretch. Therefore, this species is exposed to different environmental conditions where a flexible behaviour is mandatory to withstand short-term unpredictable changes in the swash zone. *E. affinis* had more specific trends and environmental preferences and its main occurrence was in mid-high levels of the beach, where a circadian rhythm related to tidal cycles is supposed to be an advantage for beach crustacean species in avoiding surface desiccation, predation or immersion [3], [4]. These species were the most abundant in all the beaches studied, and the highest occurrences were once again related to the food-enriched western coast.

In some cases, the models derived from different beaches revealed similar species occurrences, indicating a strong influence of a specific variable on local processes. This means that relationships produced over any of those beaches may provide a gross qualitative indication of the abiotic effect [13]. Almost all the species were sometimes directly limited by a specific variable, and in these occasions data from any beach location should provide enough information to determine the specific species’ general reaction to the variable measured. This situation can be extrapolated to other parts of the world, since the intermediate beach is the most common beach type [3]. The results obtained in this study revealed a mixed response to the predictor variables over the two contrasting scales, so important information can be gathered from examining differences between BRT models at different scales in order to build general beach species-environment relationships.

In summary, beach species dwell in a highly demanding ecosystem controlled by abiotic factors, but evidence of the importance of external food availability was shown. Our results also revealed that the local scale is a feasible way to construct general predictive species-environmental models, since relationships derived from different beaches predicted similar responses for certain macroinvertebrate species. However, additional information on aspects of specific species distribution and abundance can be derived by checking models at larger scales. Actual studies (e.g., [11], [14], [34]), including the present one, showed contrary evidence to the traditionally concept of exposed beaches as only physically stressed environments with negligible influence of the biotic compound. Finally, BRT added insight elucidating the importance of interactions between predictors for identifying the relative contributions of the predictors and species responses. This technique has proven useful for exploration, explanation and prediction offering both opportunities and challenges to sandy beach ecologists.

## Supporting Information

Figure S1 **Boxplots representing the main beach variables (mean ± standard deviation) from the northern Spanish coastline.** Mean grain size (MGS), sediment water content and temperature (T), exposure to the air (ET), morphodynamic Dean index, shear strength (SS), species richness and exposure rate (ER). The dots represent outliers. See Figure 1 for beach names (n  = 39) and Table S1 for further explanation on the variables.
(TIF)

Table S1 **Environmental variables measured across the geographic beach continuum from the Spanish northern shoreline.** Description, range of values, units, and main references are included for better comprehension.
(DOC)

Table S2 **Main species captured in the 39 exposed beaches from the North coast of Spain.** (Bp: *Bathyporeia pelagica*, Dt: *Donax trunculus*, Ea: *Eurydice affinis*, Ep: *E. pulchra*, Gs: *Gastrosaccus sanctus*, Ha: *Haustorius arenarius*, Nc: *Nephtys cirrosa*, Ob: *Ophelia bicornis*, Pa: *Pontocrates arenarius*, Ss: *Scolelepis squamata*, Sr: *Sphaeroma rugicauda*; Ts: *Talitrus saltator*; Tb: *Talorchestia brito*, Te: *Tylos europaeus*).
(DOC)

Table S3 **List of the beach species captured in the 39 exposed beaches from the North coast of Spain.** This list shows the name and taxonomic composition of 59 truly sandy beach species. We also collected 13 species of uncertain origin, mainly insects: Aranei (1 species), Coleoptera (9), Diptera (2), and Hymenoptera (1).
(DOC)
