# Supplementary figures and images for: Exploring Macroinvertebrate Species Distributions at Regional and Local Scales across a Sandy Beach Geographic Continuum
Source: PLoS One. 2012 Jun 25;7(6):e39609. doi: 10.1371/journal.pone.0039609 (PMC3382464; doi:10.1371/journal.pone.0039609)

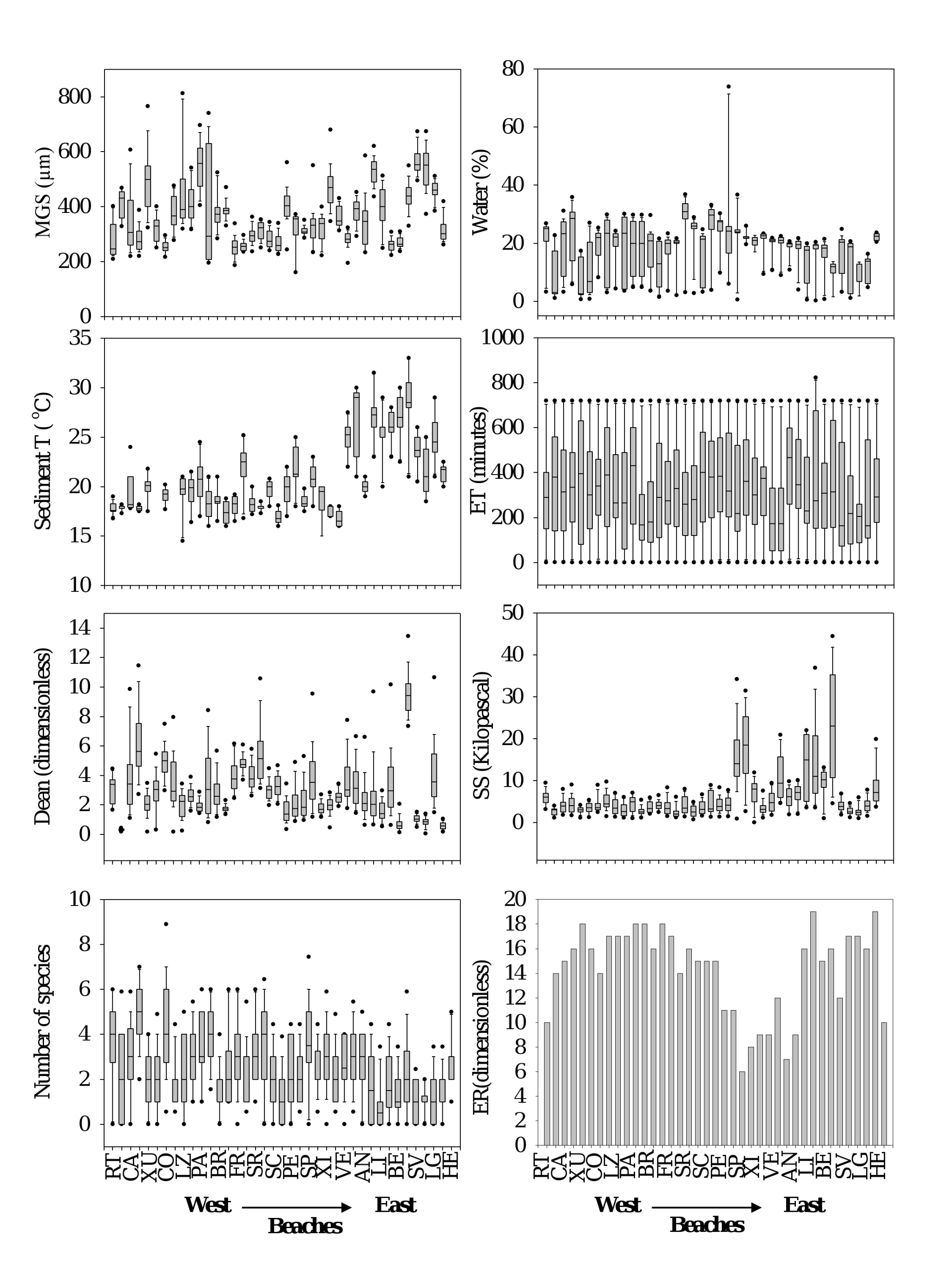

Supplement: Figure S1 — Boxplots representing the main beach variables (mean ± standard deviation) from the northern Spanish coastline. Mean grain size (MGS), sediment water content and temperature (T), exposure to the air (ET), morphodynamic Dean index, shear strength (SS), species richness and exposure rate (ER). The dots represent outliers. See Figure 1 for beach names (n = 39) and Table S1 for further explanation on the variables. (TIF) [file pone.0039609.s001.tif]
