# Supplementary material for: Exploring Macroinvertebrate Species Distributions at Regional and Local Scales across a Sandy Beach Geographic Continuum
Source: PLoS One. 2012 Jun 25;7(6):e39609. doi: 10.1371/journal.pone.0039609 (PMC3382464; doi:10.1371/journal.pone.0039609)
Supplement: Table S1 — Environmental variables measured across the geographic beach continuum from the Spanish northern shoreline. Description, range of values, units, and main references are included for better comprehension. (DOC) [file pone.0039609.s002.doc]

| Variable | Code | Description | Range^a^ | Units | Reference |
| --- | --- | --- | --- | --- | --- |
| Longitude | long | Geographic longitude (East to West). 0 is the eastern location | 0-450^b^ | dimensionless | [14] |
| Sea surface temperature* | sst | The temperature is the annual range: maximum, minimum and mean | -0.03-0.29 | ºC | [22]^c^ |
| Chlorophyll *a** | chl *a* | Chl*a* concentration calculated as the annual range | 0.44-33.4 | mg/m^3^ | [22]^c^ |
| Width | - | Beach distance across-shore (from above the drift line to the low swash zone) | 30-260 | meters | [3,8,14] |
| Length | - | Beach distance along-shore. | 240-7000 | meters | [3,8,14] |
| Slope* | S | This was determined by Emery’s profiling technique and calculated dividing the beach height by the distance between two points. | 0.01-0.05 | dimensionless | [8,14,41] |
| Wave height | Hb | Height of the breaking waves. Wave height was estimated by measuring 30 breaking waves with graduated poles against the horizon. | 0.26-2.25 | meters | [3,8,14] |
| Wave period | Tb | Period between two waves | 4.5-17.3 | seconds | [3,8,14] |
| Temperature | T | Temperature in the sediment. | 16.7-27.5 | ºC | Rodil unpublished |
| Water | W | Sediment water content (% of sediment water at each beach level). | 7.6-27.2 | % | [3,8,14] |
| Exposure time | ET | Air exposure time (time between two consecutive tides). | 0-720 | minutes | Rodil unpublished |
| Shear strength* | SS | Measure sediment compacting force using a hand held shear vane tester (Pilcon) | 2.4-23 | Kilopascal | Rodil unpublished |
| Mean grain size* | MGS | Sediment grain size using a Coulter LS 200 laser diffraction particle size analyser and the coarser fraction (> 2 mm) by dry sieving | 252 -564 | μm | [3,8,14] |
| Relative tide range | RTR | RTR = TR/Hb, where TR is spring tide range. Indicates the relative importance of tides versus waves in controlling morphodynamics. | 1.57-4.40 | meters | [8,14,42] |
| Dean parameter | Ω | It is a measure of how reflective or dissipative a beach is. Ω = ((Hb/Ws)* Tb, where Hb is breaker height (m), Ws is sand fall velocity (m s^-1^) and Tb is wave period (s). Ω < 2 characterise reflective, Ω > 5 dissipative and 2 < Ω < 5 intermediate beach. | 0.31-5.41 | dimensionless | [8,14,43] |
| Beach state Index | BSI | BSI is Ω multiplied by tide range and indicates the ability of waves and tides to move sand. < 0.5 reflective, 0.5-1 low to medium energy intermediate, 1.0-1.5 high-energy intermediate dissipative, 1.5-2.0 dissipative, and > 2.0 ultra-dissipative macrotidal beaches. | 0.37-1.67 | dimensionless | [3,6,8,14] |
| Beach Index | BI | BI = log_10_ (Sand*TR)/S where Sand is the mean grain size (phi units + 1), TR is the maximum spring tide range (m) and S is beach slope. BI < 1.5 microtidal reflective; > 3 macrotidal dissipative beaches. | 0.7-2.75 | log phi*meters | [3,8,14] |
| Exposure rate | ER | The 20-point rating system is used to estimate the wave exposure rate, which takes into account observations of wave action, median particle diameter, slope, depth of the redox potential layer and the presence of macrofaunal structures (0 is the less exposed situation). | 10.0-19.0 | dimensionless | [3,8,14,21] |

*Variables included in the regional and local final models.

^a^Minimum and maximum values.

^b^Geographical longitude standardized by subtracting the minimum value to each longitudinal point.

^c^Sea surface temperature and cholorphyll *a* metrics from http://www.oracle.ugent.be/

41. Emery KO (1961) A simple method of measuring beach profiles. Limnol and Oceanogr 6: 90-93.

42. Masselink G, Short AD (1993) The effect of tide range on beach morphodynamics and morphology: a conceptual beach model. J Coastal Res 9(3): 785-800.

43. Short AD, Wright LD (1983) Physical variability of sandy beaches. In: McLachlan A, Erasmus T. (Eds.). Sandy Beaches as Ecosystems. Junk, The Hague. 133-144.
