# Supplementary material for: Exploring Macroinvertebrate Species Distributions at Regional and Local Scales across a Sandy Beach Geographic Continuum
Source: PLoS One. 2012 Jun 25;7(6):e39609. doi: 10.1371/journal.pone.0039609 (PMC3382464; doi:10.1371/journal.pone.0039609)
Supplement: Table S2 — Main species captured in the 39 exposed beaches from the North coast of Spain. (Bp: Bathyporeia pelagica, Dt: Donax trunculus, Ea: Eurydice affinis, Ep: E. pulchra, Gs: Gastrosaccus sanctus, Ha: Haustorius arenarius, Nc: Nephtys cirrosa, Ob: Ophelia bicornis, Pa: Pontocrates arenarius, Ss: Scolelepis squamata, Sr: Sphaeroma rugicauda; Ts: Talitrus saltator; Tb: Talorchestia brito, Te: Tylos europaeus). (DOC) [file pone.0039609.s003.doc]

|  |  |  | Main species | | | | | | | | | | | | | |
| --- | --- | --- | --- | --- | --- | --- | --- | --- | --- | --- | --- | --- | --- | --- | --- | --- |
| Beach | Code | Longitude | *Bp* | *Dt** | *Ea** | *Ep** | *Gs* | *Ha* | *Nc* | *Ob* | *Pa** | *Ss** | *Sr** | *Ts* | *Tb** | *Te* |
| Rostro | RT | 449 | 0 | 0 | 0 | 168 | 31 | 0 | 0 | 0 | 20 | 2 | 0 | 19 | 6 | 0 |
| Area Longa | AL | 445 | 0 | 0 | 0 | 62 | 2 | 0 | 0 | 0 | 12 | 13 | 56 | 7 | 0 | 0 |
| Carnota | CA | 440 | 14 | 85 | 136 | 59 | 23 | 8 | 0 | 3 | 35 | 188 | 20 | 6 | 0 | 0 |
| Louro | LO | 439 | 0 | 1 | 0 | 9 | 8 | 0 | 4 | 0 | 6 | 139 | 1 | 15 | 17 | 0 |
| Xuño | XU | 437 | 0 | 0 | 2 | 15 | 13 | 0 | 0 | 0 | 28 | 2 | 0 | 3 | 35 | 3 |
| Traba | TR | 436 | 1 | 0 | 15 | 41 | 34 | 1 | 0 | 0 | 14 | 19 | 0 | 0 | 0 | 0 |
| Corrubedo | CO | 436 | 0 | 6 | 14 | 259 | 16 | 0 | 5 | 0 | 45 | 407 | 15 | 3 | 37 | 0 |
| Rodas | RO | 428 | 25 | 0 | 0 | 448 | 15 | 4 | 0 | 0 | 47 | 12 | 0 | 4 | 68 | 3 |
| Lanzada | LZ | 426 | 13 | 4 | 35 | 556 | 2 | 11 | 15 | 0 | 8 | 180 | 3 | 2 | 0 | 0 |
| Seiruga | SE | 425 | 0 | 0 | 10 | 108 | 2 | 0 | 0 | 0 | 48 | 20 | 17 | 7 | 3 | 0 |
| America | PA | 423 | 0 | 65 | 138 | 24 | 1 | 2 | 3 | 0 | 22 | 360 | 110 | 0 | 0 | 0 |
| Baldaio | BA | 415 | 130 | 0 | 1 | 105 | 30 | 0 | 10 | 40 | 23 | 190 | 2 | 0 | 0 | 0 |
| Barrañán | BR | 406 | 0 | 0 | 0 | 23 | 4 | 0 | 0 | 0 | 96 | 8 | 2 | 0 | 0 | 0 |
| Doniños | DO | 393 | 0 | 0 | 5 | 53 | 9 | 0 | 1 | 0 | 46 | 2 | 0 | 23 | 7 | 40 |
| Frouxeira | FR | 383 | 10 | 0 | 16 | 882 | 1 | 8 | 2 | 28 | 53 | 71 | 2 | 0 | 0 | 0 |
| Bares | EB | 357 | 0 | 0 | 4 | 70 | 3 | 11 | 2 | 1 | 41 | 9 | 1 | 6 | 1 | 6 |
| San Román | SR | 351 | 0 | 0 | 62 | 282 | 16 | 1 | 6 | 1 | 49 | 110 | 9 | 2 | 2 | 6 |
| Llas | LL | 329 | 5 | 0 | 1 | 15 | 0 | 0 | 0 | 0 | 95 | 19 | 18 | 9 | 0 | 10 |
| San Cosme | SC | 325 | 0 | 0 | 13 | 12 | 1 | 1 | 1 | 141 | 1 | 2 | 3 | 9 | 1 | 8 |
| Viveiro | EV | 317 | 0 | 0 | 6 | 43 | 34 | 2 | 5 | 3 | 38 | 11 | 13 | 5 | 5 | 10 |
| Peñarronda | PE | 313 | 0 | 0 | 1 | 40 | 1 | 5 | 9 | 0 | 16 | 43 | 54 | 3 | 0 | 0 |
| Otur | OT | 289 | 0 | 0 | 13 | 59 | 4 | 1 | 2 | 7 | 25 | 17 | 1 | 0 | 0 | 0 |
| San Pedro | SP | 267 | 0 | 0 | 47 | 167 | 0 | 98 | 7 | 0 | 18 | 119 | 36 | 0 | 0 | 0 |
| Xagó | XA | 249 | 0 | 0 | 24 | 458 | 7 | 6 | 1 | 0 | 0 | 2 | 1 | 8 | 0 | 5 |
| Xivares | XI | 237 | 0 | 0 | 218 | 233 | 13 | 13 | 1 | 0 | 37 | 21 | 33 | 0 | 0 | 0 |
| Espasa | ES | 207 | 0 | 1 | 12 | 152 | 13 | 1 | 0 | 98 | 14 | 3 | 18 | 4 | 0 | 0 |
| Vega | VE | 202 | 0 | 0 | 108 | 127 | 17 | 13 | 3 | 85 | 0 | 5 | 2 | 0 | 3 | 4 |
| Toranda | TO | 185 | 30 | 0 | 29 | 27 | 0 | 36 | 0 | 0 | 3 | 14 | 60 | 12 | 0 | 0 |
| Andrín | AN | 176 | 0 | 0 | 69 | 68 | 11 | 6 | 0 | 0 | 49 | 20 | 4 | 0 | 0 | 0 |
| Oyambre | OY | 154 | 3 | 5 | 31 | 49 | 11 | 59 | 1 | 4 | 2 | 10 | 1 | 0 | 0 | 0 |
| Liencres | LI | 133 | 0 | 0 | 1 | 3 | 14 | 0 | 1 | 24 | 1 | 0 | 1 | 0 | 0 | 0 |
| Langre | LN | 116 | 0 | 0 | 2 | 15 | 30 | 0 | 0 | 11 | 17 | 3 | 2 | 2 | 1 | 0 |
| Berria | BE | 101 | 9 | 6 | 3 | 4 | 3 | 8 | 0 | 0 | 4 | 3 | 1 | 0 | 0 | 0 |
| Laredo | LA | 100 | 10 | 109 | 0 | 0 | 1 | 2 | 22 | 0 | 19 | 1 | 0 | 0 | 0 | 0 |
| Salvaje | SV | 74 | 0 | 0 | 0 | 544 | 0 | 0 | 0 | 0 | 60 | 0 | 0 | 0 | 0 | 0 |
| Bakio | BK | 62 | 0 | 0 | 0 | 88 | 1 | 0 | 0 | 0 | 14 | 0 | 2 | 0 | 0 | 0 |
| Laga | LG | 53 | 0 | 0 | 0 | 22 | 12 | 0 | 0 | 3 | 11 | 8 | 0 | 0 | 1 | 0 |
| Zarautz | ZA | 23 | 0 | 0 | 0 | 223 | 11 | 0 | 0 | 0 | 5 | 3 | 0 | 0 | 0 | 0 |
| Hendaya | HE | 0 | 11 | 23 | 1 | 18 | 2 | 23 | 1 | 0 | 13 | 84 | 19 | 0 | 0 | 0 |
| Abundance |  | - | 261 | 305 | 1017 | 5531 | 396 | 320 | 102 | 449 | 1035 | 2120 | 507 | 149 | 187 | 95 |
| Presence |  | - | 12 | 10 | 29 | 38 | 35 | 23 | 21 | 14 | 37 | 36 | 30 | 20 | 14 | 10 |

Abundance is the number of the individuals in each beach. Presence is the number of beaches where the species was found. Longitude (standardized): beaches from West (449) to East (0).

*Species used in the final BRT models (Figures 4-6).
