# Supplementary material for: Exploring Macroinvertebrate Species Distributions at Regional and Local Scales across a Sandy Beach Geographic Continuum
Source: PLoS One. 2012 Jun 25;7(6):e39609. doi: 10.1371/journal.pone.0039609 (PMC3382464; doi:10.1371/journal.pone.0039609)
Supplement: Table S3 — List of the beach species captured in the 39 exposed beaches from the North coast of Spain. This list shows the name and taxonomic composition of 59 truly sandy beach species. We also collected 13 species of uncertain origin, mainly insects: Aranei (1 species), Coleoptera (9), Diptera (2), and Hymenoptera (1). (DOC) [file pone.0039609.s004.doc]

| **Species names** |
| --- |
| **Phylum Nemertea** |
| Nemertea indet |
| **Phylum Annelida**  **Class Clitellata** |
| Oligochaeta indet |
| **Class Polychaeta** |
| **Subclass Aciculata** |
| Order Eunicida |
| Family Lumbrineridae |
| *Lumbrineris tetraura* (Schmarda, 1861) |
| Order Phyllodocida |
| Family Aphroditoidae |
| *Harmothoe* sp Kinberg, 1856 |
| Family Nepthyidae |
| *Nephtys cirrosa* (Ehlers, 1868) |
| Family Phyllodocidae |
| *Eteone longa* (Fabricius, 1780) |
| *Phyllodoce* sp Lamarck, 1818 |
| *Pisione remota* (Southern, 1914) |
| Family Sigalionidae |
| *Sigalion mathildae* Audouin & Milne Edwards in Cuvier, 1830 |
| Family Syllidae |
| *Odontosyllis* sp Claparède, 1863 |
| *Syllis* sp Lamarck, 1818 |
| *Sphaerosyllis bulbosa* (Southern, 1914) |
| **Subclass Scolecida** |
| Order Ophelida |
| Family Opheliidae |
| *Ophelia bicornis* Savigny in Lamarck, 1818 |
| *Ophelia neglecta* Schneider, 1892 |
| Order Orbiniida |
| Family Orbiniidae |
| *Scoloplos armiger* (Müller, 1776) |
| Family Paraonidae |
| *Paraonis fulgens* (Levinsen, 1884) |
| **Subclass Canalipalpata** |
| Order Spionida |
| Family Spionidae |
| *Dispio uncinata* Hartman, 1951 |
| *Malacoceros fuliginosus* (Claparède, 1869) |
| *Scolelepis (Scolelepis) pettiboneae* Maciolek, 1986 |
| *Scolelepis squamata* (Müller, 1806) |
| *Scolelepis* sp. (Blainville, 1828) |
| *Spio filicornis* (Müller, 1776) |
| *Spiophanes bombyx* (Claparède, 1870) |
| **Subclass Polychaeta incertae sedis** |
| Family Saccocirridae |
| *Saccocirrus* sp Bobretzky, 1872 |
| **Phylum Mollusca; Class Bivalvia; Subclass Heterodonta** |
| Order Veneroida |
| Family Donacidae |
| *Donax trunculus* Linnaeus, 1758 |
| Family Tellinidae |
| *Tellina tenuis* da Costa, 1778 |
| **Phylum Arthropoda; Subphylum Crustacea; Class Malacostraca** |
| **Superorder Peracarida** |
| Order Amphipoda |
| Family Aoridae |
| *Aora typica* Krøyer, 1845* |
| Family Atylidae |
| *Atylus guttatus* (Costa, 1851)* |
| *Atylus swammerdami* (Milne-Edwards, 1830) |
| Family Haustoriidae |
| *Haustorius arenarius* (Slabber, 1769) |
|  |
|  |
|  |
| Family Isaeidae |
| *Microprotopus* sp Norman, 1867 |
| Family Oedicerotidae |
| *Pontocrates altamarinus* (Bate & Westwood, 1862) |
| *Pontocrates arenarius* (Bate, 1858) |
| Family Pontoporeiidae |
| *Bathyporeia pelagica* (Bate, 1856) |
| *Bathyporeia* sp Lindstrom, 1855 |
| Family Talitridae |
| *Talitrus saltator* (Montagu, 1808) |
| *Talorchestia brito* Stebbing, 1891 |
| *Talorchestia deshayesi* (Audouin, 1826) |
| Family Urothoidae |
| *Urothoe brevicornis* Bate, 1862 |
| *Urothoe poseidonis* Reibish, 1905 |
| *Urothoe pulchella* (Costa, 1853) |
| Order Cumacea |
| Family Bodotriidae |
| *Cumopsis fagei* Bacescu, 1956 |
| *Eocuma dollfusi* Calman, 1907 |
| *Cumopsis* sp. (G.O. Sars, 1865) |
| Order Isopoda |
| Family Cirolanidae |
| *Eurydice affinis* Hansen, 1905 |
| *Eurydice pulchra* Leach, 1815 |
| *Eurydice spinigera* Hansen, 1890 |
| Family Sphaeromatidae |
| *Lekanesphaera hookeri* (Leach, 1814) |
| *Lekanesphaera rugicauda* (Leach, 1814) |
| Family Tylidae |
| *Tylos europaeus* Arcangeli, 1938 |
| Order Mysida |
| Family Mysidae |
| *Gastrosaccus sanctus* (van Beneden, 1861) |
| *Gastrosaccus spinifer* (Goës, 1864) |
| *Haplostylus normani* (G.O. Sars, 1877)^c^ |
| **Superorder Eucarida** |
| Order Decapoda |
| Family Diogenidae |
| *Diogenes pugilator* (Roux, 1829) |
| Family Portunidae |
| *Portumnus latipes* (Pennant, 1777) |
| **Phyllum Chordata; Subphyllum vertebrata** |
| **Class Actinopterygii** |
| Order Perciformes |
| Family Ammoditidae |
| *Ammodytes tobianus* Linnaeus, 1758 |
| Family Trachinidae |
| *Echiichthys vipera* (Cuvier, 1829) |
| Order Pleuronectiformes |
| Family Scophthalmidae |
| *Psetta maxima* (Linnaeus, 1758) |
